# South West Surgical Club

**Published:** 1992-08

**Authors:** 


### South West Surgical Club

### Meeting held at Bristol Zoo Conference Suite 18 October 1991

**THYROID AUDIT 1988-1990**
R. E. May

A review of 3 years in-patient thyroid surgery was undertaken to assess presentation diagnosis and morbidity. 63 goitres and 3 thyroglossal cysts were admitted under the author. The mean age at presentation was 43 years and 80% were female. 71% of the patients had a symptomless, solitary swelling, 16% a diffuse goitre and 13% thyrotoxicosis.

The ultra-sound scan has been the most useful diagnostic tool. Fine needle aspiration cytology was used on all solitary swellings but it failed to confirm malignancy in the 7 thyroid carcinomata. Some points were stressed about surgical tecniques, in particular the necessity for care in tying the superior thyroid vessels and so avoid damage to the external laryngeal nerve. The author felt it important to identify the recurrent laryngeal nerve in all operations. There were no cases of nerve damage in this series, there was 1 minor wound infection. Solid thyroid lesions should be excised by Ipsilaterial Total Lobectomy. Malignancy has to be confirmed histologically prior to proceeding to Total Thyroidectomy. This may be delayed until paraffin sections have been examined at 48hrs. Re-exloration of the neck on the contra lateral side was carried out with difficulty but there was 1 case of post operative hypoparathyroidism.

**MEDICAL TREATMENT OF INTESTINAL OBSTRUCTION**
Ian Capstick
St. Peter's Hospice

The management of intestinal obstruction, which is secondary to carcinomatosis presents doctors and nurses with a formidable challenge. Most patients have passed the stage where curative procedures can be considered and even palliative options are limited.

Surgical intervention should be considered in all patients who are or are about to obstruct. The actual number of patients in whom surgery is a possibility and where the patient agrees will be small (2 out of 40 patients; M. Baines et al; accepted for publication Lancet) the factors to be considered are:

1) Is there likely to be a single, removable lesion?
2) Is the general condition of the patient such that surgery is an option?
3) If there was previous surgery, what were the operative findings?
4) What does the patient think about surgery?

Most patients will opt for medical management if given the choice. On balance, when offering surgical intervention the hospice doctor and surgeon have not only to fulfil the above criteria but of course they must have the reasonable chance of improving the quality of the patient's remaining life.

**Medical Management**

This remains the lynch-pin of care. The group of symptoms presenting in intestinal obstruction are some of the most distressing and very often abdominal colic, abdominal pain with nausea, and vomiting can be present at the same time in the same patient.

Explaining to patient and family as to what is happening, why, and how it is to be managed is crucial. Very often it is the unspoken worry or question that needs to be answered. "Will my tummy burst"? "Is this sickness never going to stop"? "I haven't had my bowels opened for days"? - are typical of the questions never asked but should be anticipated when setting goals for management.

**Drip and suck.**If surgical intervention is actually a possibility our patients are rehydrated and managed with IV fluids and naso-gastric tubes, in the surgical unit involved. As a general rule we do not either 'drip or suck' patients in the hospice - this is a policy accepted by medical and nursing staff. There are occasions when it seems as if it would be reasonable to drip patients - particularly with packed cells - but they are few and far between. Most patients find nasogastric tubes uncomfortable and if given the choice the majority would prefer to do without them.

**Hydration.** Acceptable levels of hydration can be achieved by the use of small amounts of fluid at frequent intervals. A variety of methods can be tried from small ice cubes to ice lollies and mineral drinks. Each unit has its own idiosyncratic ideas on fluid intake.

**Syringe Driver.** Continuous infusion by the subcutaneous route using a syringe driver has been a welcome addition in the management of abdominal pain and colic, together with nausea and vomiting. Using this method peaks and troughs of circulating drugs are abolished; in addition there is no longer any necessity for regular 4 hourly intra muscular injections, either in the in-patient unit or in the home setting.

**Intestinal colic.** The intestinal gripping or 'griping'pains of colic are best treated with smooth-muscle relaxant drugs; loperamide (immodium), hyoscine or atropine. They can be given orally (or sublingually but a substantial proportion will require medication subcutaneously using a syringe driver. In a few patients a coliac axis block, carried out by an anaesthetist can produce significant relief from colic.

**Abdominal pain.** This occurs in the majority of patients and is distinct from the intermittent pain of intestinal colic, in that it is continuous. Most patients can expect to be well controlled, although the level of medication needs to be constantly monitored and assessed. The majority will receive opiates by one route or another. Pain from hepatomegaly can sometimes be helped by the addition of steroids to the analgesia (6 mg Dexamethasone daily). When oral medication is precluded oxycodone suppositories can be a useful alternative, however, some patients dislike suppositories and in others proctitis prevents continulng use. One oxycodone suppository is equivalent to 30 mg of oral morphine.

**Nausea & Vomitinq**. The majority, if not all patients will suffer from nausea and vomiting. It is the most troublesome of symptoms to abolish. Mary Baines achieved relief of nausea and vomiting in 13% of patients, llX continued to have moderate to severe problems and 76X mild symptoms (defined as one or less vomits per day with little or no nausea).

**Anti-Emetics**

| | | |
|---|---|---|
| Prochlorperazine | (Stemetil) | orally, suppository or IM Injection |
| Chlorpromazine | (Largactil) | |
| Cyclizine | | orally or IM Injection |
| Methotrimeprazine | (Nozinan) | by subcutaneous, continuous infusion |
| Haloperidol | (Serenace) | |
| Cyclizine | (Valoid) | |

Quite a few patients will continue to vomit a meal having previously enjoyed the eating of it. Some patients in fact will choose this episodic pattern of vomiting.

**Diarrhoea**. The use of conventional anti-diarrhoea drugs such as loperamide (immodium diphenoxylate (lomotil) and codeine will help in these circumstances. Fistulae can be a most distressing addition as it often makes total control of the diarrhoea impossible. The diarrhoea of partial or subacute obstruction can sometimes continue until death, and precedes complete obstruction.

**Constipation.** Adominal palpation of the descending colon together with repeated rectal examination establishes this diagnosis. Faecal softeners such as docusate ( dioctyl) or arachis oil enemas are used for this. Constipation can be anticipated and prevented whenever moderate or strong analgesics are used. Both metoclopramide (maxolon) and danthron (dorbanex) increase small bowel peristalsis which in turn can increase abdominal colic and pain - they are best avoided in these circumstances.

### TRAUMA - A GUIDE TO MANAGEMENT

C. Forrester-Wood, F.R.C.S.
Consultant Thoracic Surgeon

Management of chest trauma starts at the roadside with recognition of the immediate life-threatening injuries. It has not been convincingly demonstrated that intervention at the roadside has any benefit with the exception of stabilising the cervical spine, ensuring the airway and possibly commencing an intravenous drip.

Different types of injury occur with high velocity, low velocity and crush injuries. Understanding the mechanism of the injury will help to recognise the possible sites of injury.

All penetrating injuries of the chest must be carefully monitored. Not all such injuries need exploration. Indications for emergency thoracotomy are: cardiac tamponade, major bronchial disruption and major haemorrhage. Indications for elective emergency thoracotomy are: continuing bleeding after insertion of chest drains in excess of 300 ccs. and hour, clotted haemothorax, ruptured diaphragm and continuing airleak with persisting collapse of the lung.

Flail chest in association with other injuries is usually treated by intubation and intermittent positive pressure ventilation. Flail chest in isolation does not necessarily require IPPV. The mainstay of treatment in such cases is adequate pain relief and mobilisation. Adequate pain relief is achieved with a thoracic epidural catheter and continuous infusion of local anaesthetic with opiates. Using this technique patients are mobilised more quickly, nutrition is better and ventilation is avoided.

### 100 SUCCESSFUL NECK EXPLORATIONS FOR TERTIARY HYPERARATHYOIDISM

Humphrey White

102 pts under treatment for chronic renal failure at Southmead Hospital underwent exploration of the neck with cinical biochemical evidence of tertiary hyperparathroidism between 1969 and 1991. Two explorations failed to find abnormal parathyroid glands. In the remaining 100 pts, between two and five hyperplastic glands were found in 87 pts, a single adenoma in 8 pts and two adenomata in 5. Two patients were discovered to have an unsuspected papillary cell carcinoma of the thyroid. All 100 patients became normocalcaemic post operatively, some with oral calcium supplents or Vitamin D analogues.

11 patients underwent re-exploration for recurrent disease after an interval of normocalcaemia varying fron 8/12 to 16 yrs. Hyperplasia of a 1/2 gland remnant was found in 5 pts and a previously undiscovered gland in 6 pts. One patient had a sixth gland removed from within the thymus at her third operation.

No patient suffered permanent recurrent laryngeal nerve damage. There were two postoperative deaths; one from a ruptured berry aneurysm and the other, undergoing unsuccessful neck operation, from a pulmonary embolus associatied with a lymphoma.

### JEJUNAL TRANSFERS FOR CARCINOMA OF PHARYNX

Mr. Paul Lear
Consultant Senior Lecturer
Southmead Hospital

Carcinoma of the pharynx occurs most frequently in elderly and malnourished patients. There is often a history of tobacco and/or alcohol abuse. The tumour presents late with dysphagia. Treatment rests between radical radiotherapy (preferred by most surgeons in the UK) and surgery which is usually reserved for those suffering recurrent disease or lymph node involvement.

Free jejunal transfer was adopted for seven patients at the Royal National Throat, Nose and Ear Hospital, London. This centre has extensive experience with gastric pull up and colonic swing procedures. The jejunal transfer was adopted because of the serious morbidity and long in-hospital stay associated with the major surgery. Three of the seven patients had undergone gastric surgery previously. Four patients required pre-operative gastrostomy/jejunostomy tube feeding for two weeks and all were fed similarly post operatively for seven days. Total laryngopharyngectomy with/without functional block dissection of the neck were carried out as for previous surgery, but the defect was bridged by a segment of mid jejunum on a single vascular pedicle. This was anastomoed to the superior thyroid artery stump and the internal jugular vein. One graft became tender three days post operatively and the patient feverish. The neck was re-explored, the graft removed and an oesophagostomy raised. He awaits further reconstruction. The remainder have had no post operative complications, and were dicharged home within 12 days swallowing well. All have remained well 6 - 18 months after surgery. Jejunal transfer provides a less radical but practical way of treating carcinoma of the pharynx surgically.

### SIMULTANEOUS RECORDING OF NASO-PHARYNGOSCOPY 7 FLOUROSCOPY

Mr. Ronald W. Pigott

In speech, the soft palate lifts to close the velopharyngeal isthmus, so that the air stream from the lungs is directed past the tongue, teeth and lips which modulate it to produce the sounds we recognise. When the isthmus fails to close due to a large number of congenital and acquired conditions, air escapes audibly through the nose and abnormal resonance is perceived. This hypernasal resonance is quickly picked up by children as ridiculous and the sufferer can become introverted and fail to achieve potential. Volume and length of utterance can be seriously diminished. Children may even end up in schools for the mentally subnormal.

To select an appropriate operation to correct this problem a technique has been developed in Bristol of simultaneous video recording of the appearance of the isthmus viewed by an endoscope passed through the nose while an image intensifier produces a lateral pharyngeal x-ray. The original equipment to combine the images was built in the university but now "off the shelf" equipment is available at a modest cost, using a Panasonic effects mixer and a Panasonic quad split unit (designed for the surveillance world) where up to 4 video camera images can be reviewed on one screen. The ability to play back and re-analyse the brief recordings obtainable within the tolerance of small children for examination is invaluable. Endoscopy provides excellent quantitative measurement and x-rays good qualitative information the two modes are complementary.

Since using this system we have been able to improve success rates for operations by nearly 20% on previously published studies.

### TALES FROM THE ORIENT

Mr. D. J. Leaper
Consultant Senior Lecturer
Southmead Hospital, Westbury-on-Trym, Bristol BS10 5NB

Between November 1988 and January 1990 I was granted leave from the University for a sabbatical in the University Department of Surgery at Queen Mary Hospital in Hong Kong. The head of department, Professor John Wong runs an impressive, very large department which embraces virtually all aspects of surgical care. As a "visiting professor" I was able to have contact with each of the departments' sub-divisions which came together every week at audit meetings and grand rounds. Such an approach has been lost to some extent in the UK and going backto a global view of surgery was fascinating .......and interesting when on my first grand rounds, was asked for my opinion on management of childhood biliary atresia, intracerebral vascular malformations and repair of stenosed trachea !

Each team was headed by a senior university surgeon. Standards were high, facilities adequate and care given to the non-paying patients (as in our NHS) was exemplary. A brand new hospital opened as I left, on the Hong Kong island site which now boasts the best facilities also. I can recommend a year's visit to anyone in training as the experience was vast. I was particularly impressed by Professor Wong's interest in oesophageal surgery and the remarkable numbers of patients who presented with the liver diseases, primary hepatocellular cancer and recurrent pyogenic cholangitis, which we rarely if ever see in the UK.

What of Hong Kong's future ? Who can tell ? It is a wonderful subtropical environment with, in my view, the most interesting view of the Orient. The contrast between downtown Central and the unspoilt northern New Territories is fascinating. Do visit before 1997 - no one knows of China's plans. Many university and medical staff are leaving but the numbers wishing to remain testify to their confidence for the Colony's future.

### DAY CASE AND OVERNIGHT STAY FOR ANAL SURGERY. A PROSPECTIVE TRIAL.

B. A. Coghlan, C. P. Armstrong and D. C. C. Bartolo
University Dept. of Surgery, Bristol Royal Infirmary, Bristol.

To evaluate the suitability of local anaesthetic and short stay admission for anal surgery sixty-nine consecutive suitable patients were entered into a prospective trial. Patients had anaesthesia by either a caudal, local infiltration or a pudendal block. The procedures performed were lateral anal sphincterotomies, haemorrhoidectomies, skin tag excisions and fistula surgery.

Full responses and follow-up at six weeks and six months was obtained in 55 patients ( 80 %) with a mean age of 48 years (range 16 to 77). 33 were day cases and 22 stayed overnight.

Pain was assessed by the patient on a four-point scale, ten patients experienced mild or moderate pain peri-operatively but only 7 required additional opiate analgesia. 21 patients received oral analgesia for post-operative pain. At home 30 patients took the prescribed analgesia for mild or moderate pain and in only 3 patients was this insufficient to relieve the pain.

Complications occurred in two patients: one patient was readmitted with postoperative bleeding and another with a deep vein thrombosis.

45/55 patients would undergo repeat anal surgery with local anaesthetic and either a day case or overnight stay. This has important implications for reducing surgical waiting lists.

### AUTOMATED SPHYGMOMANOMETERS FOR MEASURING ANKLE PRESSURE

Dr. D.H. Bennett, Dr M. Hendrick, Mrs J.A. St. Johnston, (Mr W.B. Campbell)

Increasing numbers of doctors are acquiring Doppler probes to diagnose lower limb arterial disease. This is not always easy for the occasional user, and a simple method independent of observer error would be an advantage. We have evaluated two inexpensive automatic digital sphygmomanometers (OMRON and OHTO), comparing the results with Doppler pressure measurements.

Thirty eight subjects were studied (29 male; age 18-90, median 67). Doppler pressure indices were used to separate limbs into 3 groups:- normal >.90 (n=35), mild arterial disease 0.50-0.89 (n=35), and severe disease <0.50 (n=6).

Both automatic machines gave clearly abnormal readings in all limbs with severe disease. For pressure indices <0.50 sensitivity and specificity were 100% and 77% for OMRON, and 83% and 81% for OHTO. The OHTO machine was best for separating limbs with arterial disease from those without (sensitivity 88%).

Three consecutive measurements were done on 15 limbs, and showed coefficients of variability for pressure index of 0.07 (OMRON) and 0.15 (OHTO). These compare favourably with the variability of Doppler.

These simple automated machines do not require the experience needed for Doppler pressure measurements and are cheaper than Doppler probes. They should be considered by non-specialists for evaluating suspected lower limb ischaemia.

### COMPUTERISED TOMOGRAPHY (CT) VERSUS MAGNETIC RESONANCE IMACING (MRI) IN THE PRE-OPERATIVE EVALUATION OF OESOPHAGEAL CARCINOMA

S.P. Curtney, R.H. Kennedy, D. Glew, B. Warren, P.R. Goddard. J.P. Virjee and D. Alderson
Departments of Surgery, Clinical Radiology and Pathology, Bristol Royal Infirmary, Bristol, BS2 8HW

Accurate pre-operative staging of oesophageal cancer is essential to avoid unnecessary surgery in patients with haematogenous metastases, to identify resectable tumours and determine the extent of lymph node (LN) involvement. CT and MRI were evaluated prospectively in 20 patients. Scans were performed and reported by independent radiologists, "blind" to the result of the other scan. Three patients with blood-borne spread did not undergo surgery. Sixteen patients underwent oesophagectomy with macroscopic clearance and one patient underwent laparotomy but was found to have omental metastases.

| | Haematogenous spread | | Histological LN status | | | Histological clearance | |
|---|---|---|---|---|---|---|---|
| CT | + | - | + | - | CT | Complete | Incomplete |
| + | 3 | 0 | 2 | 0 | Resectable | 12 | 2 |
| - | 1 | 16 | 8 | 6 | Unresectable | 1 | 1 |
| Sens | 75% | | 20% | | | 92% | |
| Spec | 100% | | 100% | | | 50% | |
| pv | 100% | | 100% | | | 86% | |
| MRI | | | | | MRI | | |
| + | 3 | 0 | 2 | 0 | Resestable | 6 | 2 |
| - | 1 | 16 | 8 | 6 | Unresectable | 7 | 1 |
| Sens | 75% | | 20% | | | 46% | |
| Spec | 100% | | 100% | | | 33% | |
| ppv | 100% | | 100% | | | 75% | |

Key — Sens = sensitivity, spec = specificity, ppv = positive predictive value.

CT sensitively predicts resectability (92%) but with poor specificity (50%). MRI overstages tumours as locally unresectable by over 50%. Neither technique reliably identifies LN metastases. The only value of CT/MRI is in detecting haematogenous spread. Prediction of LN status and resectablity require alternative approaches.

